# Estimation of central blood pressure waveform from femoral blood pressure waveform by blind sources separation

**DOI:** 10.3389/fcvm.2023.1280899

**Published:** 2023-11-16

**Authors:** Laila Gbaoui, Christoph Hoeschen, Eugenijus Kaniusas, Saher Khatib, Stephan Gretschel, Ernst Wellnhofer

**Affiliations:** ^1^ Chair of Medical System Technology, Institute for Medical Instrumentation, Otto von Guericke University, Magdeburg, Germany; ^2^ Institute of Biomedical Electronics, Vienna University of Technology (TU Wien), Vienna, Austria; ^3^ Department of General, Visceral-, Thoracic and Vascular Surgery, University Hospital of Ruppin-Brandenburg, Neuruppin, Germany; ^4^ Faculty of Health Sciences Brandenburg, Brandenburg Medical School Fontane, Neuruppin, Germany; ^5^ Institute of Computer-Assisted Cardiovascular Medicine, Charité, Corporate Member of Freie Universität Berlin and Humboldt Universität zu Berlin, Berlin, Germany

**Keywords:** aortic blood pressure, blind source separation analysis, single-channel independent component analysis, femoral artery, pulse wave propagation, state space reconstruction, pulse wave reflection, central blood pressure estimation

## Abstract

**Background:**

Central blood pressure (*cBP*) is a better indicator of cardiovascular morbidity and mortality than peripheral BP (*pBP*). However, direct *cBP* measurement requires invasive techniques and indirect *cBP* measurement is based on rigid and empirical transfer functions applied to *pBP*. Thus, development of a personalized and well-validated method for non-invasive derivation of *cBP* from *pBP* is necessary to facilitate the clinical routine. The purpose of the present study was to develop a novel blind source separation tool to separate a single recording of *pBP* into their pressure waveforms composing its dynamics, to identify the compounds that lead to pressure waveform distortion at the periphery, and to estimate the *cBP*. The approach is patient-specific and extracts the underlying blind pressure waveforms in *pBP* without additional brachial cuff calibration or any *a priori* assumption on the arterial model.

**Methods:**

The intra-arterial femoral *BP*_fe_ and intra-aortic pressure *BP*_ao_ were anonymized digital recordings from previous routine cardiac catheterizations of eight patients at the German Heart Centre Berlin. The underlying pressure waveforms in *BP*_fe_ were extracted by the single-channel independent component analysis (SCICA). The accuracy of the SCICA model to estimate the whole c*BP* waveform was evaluated by the mean absolute error (MAE), the root mean square error (RMSE), the relative RMSE (RRMSE), and the intraclass correlation coefficient (ICC). The agreement between the intra-aortic and estimated parameters including systolic (SBP), diastolic (DBP), mean arterial pressure (MAP), and pulse pressure (*PP*) was evaluated by the regression and Bland–Altman analyses.

**Results:**

The SCICA tool estimated the c*BP* waveform non-invasively from the intra-arterial *BP*_fe_ with an MAE of 0.159 ± 1.629, an RMSE of 5.153 ± 0.957 mmHg, an RRMSE of 5.424 ± 1.304%, and an ICC of 0.94, as well as two waveforms contributing to morphological distortion at the femoral artery. The regression analysis showed a strong linear trend between the estimated and intra-aortic SBP, DBP, MAP, and PP with high coefficient of determination *R*^2^ of 0.98, 0.99, 0.99, and 0.97 respectively. The Bland–Altman plots demonstrated good agreement between estimated and intra-aortic parameters with a mean error and a standard deviation of difference of −0.54 ± 2.42 mmHg [95% confidence interval (CI): −5.28 to 4.20] for SBP, −1.97 ± 1.62 mmHg (95% CI: −5.14 to 1.20) for DBP, −1.49 ± 1.40 mmHg (95% CI: −4.25 to 1.26) for MAP, and 1.43 ± 2.79 mmHg (95% CI: −4.03 to 6.90) for PP.

**Conclusions:**

The SCICA approach is a powerful tool that identifies sources contributing to morphological distortion at peripheral arteries and estimates *cBP*.

## 1. Introduction

**Figure 1:**
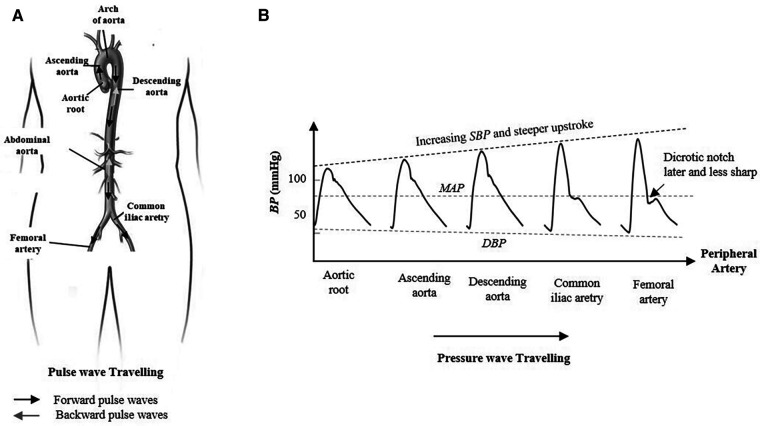
Morphological change in the arterial pressure as the contraction pulse wave travels from aortic root toward the periphery. (**A**) Pulse wave traveling in the arterial system between the aortic root, femoral artery, and iliac artery. The forward wave travels from the ventricle to the periphery of the arterial tree and the backward wave travels in the reverse direction. (**B**) The pulse pressure amplification and the systolic blood pressure increase due to pulse wave propagation and transmission from root to the periphery. In contrary, the mean arterial pressure and the diastolic blood pressure are nearly constant along the arterial system. The incisura of the central aorta pulse is replaced by a relatively later dicrotic notch that occurs at lower pressure levels.

Central blood pressure (*cBP*) is generally a superior source of information on cardiac dynamics and global circulation than peripheral blood pressure (*pBP*) (1, 2). The direct acquisition of *cBP* at the ascending aorta implies cardiac catheterization that is highly invasive, costly, time consuming, and requires skilled physicians. Thus, this procedure is unsuitable for routine screening in clinical settings. Regrettably, *pBP* cannot be used as a direct surrogate for the *cBP* due to varying and individual arterial stiffness, pulse wave propagation, and reflection along the arterial system (3), all leading to distorted pulse wave morphology between aortic root and peripheral arteries, as illustrated in Figure 1.

The brachial cuff sphygmomanometer is widely used as a routine and non-invasive measurement of BP in clinical use. However, the cuff brachial BP (*bBP*) is a poor surrogate for *cBP*. The systolic *bBP* can exceed the central systolic pressure *cSBP* up to 40 mmHg (4, 5). The *bBP* is accepted in clinical settings as a surrogate of outcome rather than corresponding intra-arterial BP (6). Accumulating evidence supported by several studies suggested that the *cBP* is strongly related to cardiovascular events and responds differently to certain vasomodulating drugs than *bBP* (7, 8).

Today, several attempts have been developed to estimate the *cBP* from the pressure waveform recorded at distal arteries such radial and carotid by applanation tonometry and calibrated to systolic, diastolic, or mean of *bBP* recorded by a cuff sphygmomanometer. Each of the proposed approaches has its own strengths and shortcomings. The carotid pressure waveform is often preferred as a direct surrogate of *cBP* due to its proximity to the aorta and is calibrated by mean and diastolic *bBP* that are assumed to be constant throughout the arterial system (4, 9). However, this technique requires highly skilled staff and is not fully integrated in the routine assessment of the *cBP* in clinical settings due to the difficulty to obtain an accurate signal in all patients, especially in obese individuals. In addition, under-estimation of the low aorto-carotid pulse pressure (*PP*) amplification can lead to over-estimation of *cBP*. Alternatively, the *cBP* can be estimated from the pressure waveform of the radial artery using either a group-averaged generalized transfer function (GTF) model (10–12), an adaptive transfer function (ATF) (13), the identification of the late systolic shoulder of the pressure waveform, or a proprietary empirical algorithm (14, 15). The different GTF approaches are based on exogenous population models that are not individualized. The ATF uses an additional pulse delay time from core to the periphery in order to personalize the model and improve the accuracy of the estimated *cBP*. However, the transfer function (TF) is still based on a population averaged model and requires a second measurement that is not necessary for TF derivation. Nowadays, a variety of instrumentations are available, which are based on one or more of these approaches (4–6). However, the main drawback of these non-invasive devices is the calibration with the brachial cuff pressure rather than intra-arterial brachial pressure that can lead to under-estimation of the true invasive pressure at the brachial artery and *cBP* (16, 17). Second, the brachial-to-radial amplification is not taken into account and can lead to additional under-estimation of *cBP*. The *cSBP* can be estimated by a simple algorithm or directly from the late systolic shoulder of invasive measured BP waveform at distal arteries without calculation of TF (8, 12, 18). However, this method has limitations, especially in younger people with non-augmented peak systolic pressure or individuals with low blood pressure (9, 19).

Alternative statistical methods for *cBP* waveform estimation comprise the blind source separation (BSS) algorithms that are subject-specific and independent of the arterial model (20–23). The approaches are widely used in biomedical signal denoising and separation (24–27) and model the arterial system as a single-input multiple-output system in order to estimate the *cBP* as an input signal that propagates through unknown channels from central to peripheral arteries using a synchronized multi-channel recording of *pBP* at specific times. The main advantage of BSS is that only the knowledge of the recorded *pBP* signals as output of the model without further assumptions on the used arterial model is required. Other researchers investigated different machine learning approaches (MLA) and neural network approaches to estimate the *cBP* from *pBP* (28, 29) or from other physiological signals such as electrocardiography (ECG) and photoplethysmography (PPG) (30). However, MLA focused mainly on the estimation of central indices such as *cSBP*.

Moreover, the pulse wave analysis is mainly focused on the derivation of indices from time and morphological characteristics defined by fiducial or inflection points on the BP waveform, as well as the area under BP waveform rather than the analysis of the pressure components that contribute to the morphology of this multivariate BP signal. Thus, in the last few decades, several approaches such as the wave separation analysis (WSA) (31), wave intensity analysis (WIA) (14, 5, 32–34), reservoir wave concept (RWC) (35–37), and instantaneous wave free ratio approach (31) have been developed to decompose *cBP* or *pBP* into their generating pressure waves. The approaches are based on different theories and arterial models. Consequently, the number and the interpretation of the extracted components depend strongly on the assumed hypothesis and the used arterial model. WSA and WIA assume that the measured pressure is a sum of a forward pressure wave generated by the left ventricle ejection and backward wave as a sum of several reflected waves from the different sites of the arterial tree system between center and periphery. The RWC assumes that the measured arterial pressure is the sum of a reservoir pressure *BP*_res_ related to the dynamical storage and release of blood by compliant arteries, and an excess pressure *BP*_exc_, which is determined by local arterial characteristics and responsible for local changes in the pulse wave. The *BP*_res_ wave varies temporally in the same way throughout the arterial system, but with a time lag that depends on the travel time from the root to the distal location, the properties of the arteries, and input from the heart (38, 39). On the other hand, all these separation techniques require several recordings, which is generally not feasible in clinical settings. WSA and RWC use simultaneous recording of pressure and flow, whereas WIA uses their derivatives. *BP*_res_ can be calculated from the pressure without the need of local flow recording, however under several additional assumptions (40).

Accordingly, it is highly desirable to estimate the subject-specific *cBP* and investigate the cardiovascular dynamics between the root and periphery without recourse to experimental population using GTF, additional measurements, or brachial cuff calibration. Thus, in order to extract the *cBP* from *pBP* waveform in a strategic way, we introduce in this study the single-channel independent component analysis (SCICA) of a single recording of *pBP* to extract the underlying pressure waveforms contributing to its dynamic and identify the compounds that cause morphological distortion and augmentation at distal arteries. The proposed SCICA in this study is a combination of the non-linear embedding of the single recording of *pBP* in a high-dimensional state space by the method of delay (41) and the BSS of the reconstructed state vectors of *pBP* in this space by the independent component analysis (ICA) (42). Moreover, the calibration of the estimated BP waveforms is based only on the wide accepted hypothesis that the mean arterial pressure (*MAP*) and diastolic pressure (*DBP*) are nearly constant in the arterial tree. The intra-arterial pressure at the femoral artery *BP*_fe_ and intra-aortic pressure *BP*_ao_ at the ascending aorta were used to validate the proposed SCICA approach.

## 2. Methods

### 2.1. Subjects and data acquisition

The study was conducted with archived fully anonymized data acquired at the German Heart Centre Berlin between 2000 and 2007 during routine cardiac catheterization by a femoral approach in eight patients providing written informed content. Simultaneous pressure recordings from the sheath connected to a pressure transducer by a fluid-filled line in the femoral artery and a catheter placed with the tip in the ascending aorta were used for *BP*_fe_ and *BP*_ao_ acquisition. In total, 32 pressure waveform recordings were acquired for this study in two raters (two *BP*_fe_ and two corresponding *BP_ao_* per subject). The pressure waveforms have been digitized at a sample rate of 500 Hz using 12-bit resolution CE certified AD interface device directly from pressure transducers. Table 1 provides a summary of biometric data.

**Table 1:** Subject characteristics.

Characteristic	Mean ± SD
***N*** (m/f)	7/1
Age (years)	34 ± 11.58
BMI (kg/m^2^)	24.73 ± 3.44
Height (cm)	176.75 ± 9.67
Weight (kg)	77.25 ± 12.04

The required *post hoc* sample size for further validation of the SCICA model was estimated to 15 subjects (17 for 10% dropout) using the interclass correlation coefficient (ICC) of 0.94 as inter-subject and intra-subject reliability index. ICC was estimated from the preliminary results of this study including Pearson's correlation between the estimated and the measured c*BP* in all patients and in both raters (43–45). The sample size calculation was performed by the calculator retrieved from https://wnarifin.github.io/ssc/ssicc.html with a minimum acceptable reliability of 0.75, a significance level of 0.05, a power of 80%, an estimated ICC of 0.94, using two measures per subject, and a dropout rate of 10%.

### 2.2. Statistical analysis and validation

All data in the present study were evaluated using the Statistical and Machine Learning Toolbox in MATLAB. The distribution of the data was investigated using the Kolmogorov–Smirnov test. The *BP*_fe_ was decomposed using SCICA and the estimated pressure compounds were identified according to their time and morphological characteristics as well as their contribution to *BP*_fe_.

#### 2.2.1. Peripheral blood pressure waveform decomposition by SCICA

ICA is a sub-class of unsupervised machine learning approaches that is widely used in the signal processing for signal denoising, dimension reduction, and blind (unobserved) sources separation techniques without any background knowledge about these hidden sources. The classical ICA model assumes that a set of *n* recorded signals $x(t)\;=\;[x_{1}(t),x_{2}(t),\ldots ,x_{n}(t){]}^{T}$ is a linear combination of *k* ≤ *n* statistical independent blind sources $s(t)\;=\;[s_{1}(t),s_{2}(t),\ldots ,\;s_{k}(t){]}^{T}$.

(1) $$x_{i}=\overset{k}{\underset{\;j=1}{\sum }}a_{ij}s_{j}\;\;\mathrm{for}\;\;i\;=\;1,\ldots ,n\;\;$$

The coefficient *a_ij_* represents the weight of the blind source *s_j_* in the recorded signal *x_i_* and form the full range *n* × *k* mixing matrix ***A***. The ICA model can be succinctly written in vector-matrix form as

(2) x(t)=A.s(t)

The ICA algorithms attempt to find a linear transformation of the blind sources and solve the blind source problem by finding a de-mixing matrix ***W*** separating the sources by only giving mixture signals *x* such that

(3) $$\hat{s}(t)\;=\;W\cdot x(t)$$

where $\hat{s}$ is the estimation of the unobserved sources s with ICA.

ICA is considered an extension of the principal component analysis that optimizes the covariance matrix of the data which represent the second order statistics. The different ICA algorithms extract the independent components by different optimization procedures including maximation of the non-Gaussianity of the sources by high order statistics (e.g., kurtosis, negentropy), minimizing the mutual information between the sources, or maximum likelihood (42, 46, 47).

**Figure 2:**
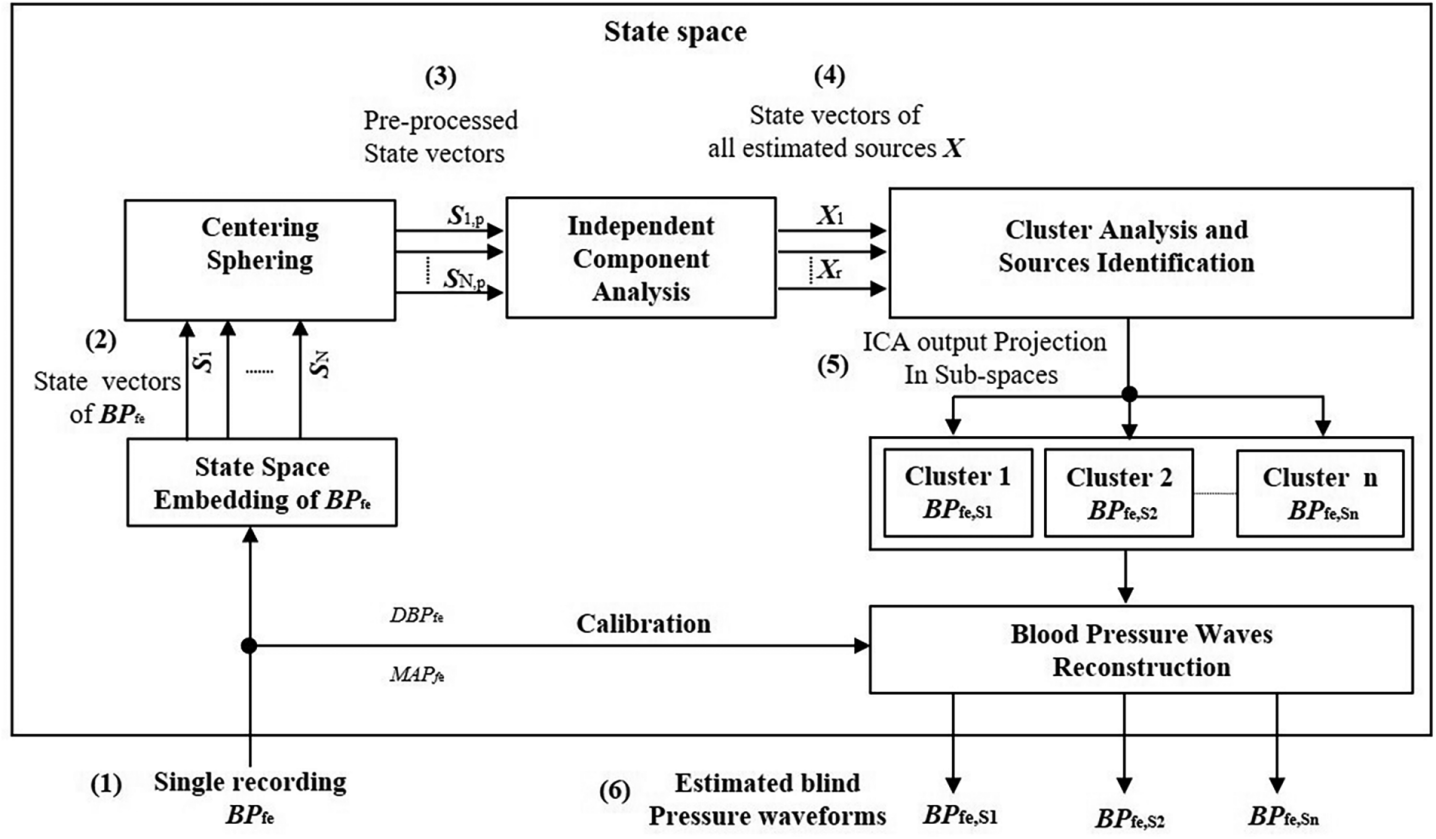
Principle of the decomposition of the single recording of the intra-arterial blood pressure waveform at femoral artery *BP*_fe_ by the SCICA: (**1**) single recording of *BP*_fe_ as input signal for the SCICA; (**2**) non-linear embedding of the *BP*_fe_ in state space using the method of delay and reconstruction of time-delayed m-dimensional state vectors (***S****_i_*, for *i *= 1, …, *N*) that capture the similar dynamics as in *BP*_fe_; (**3**) centering and whitening of state vectors (***S****_i_*_,*p*_, for *i *= 1, …, *N*) of *BP*_fe_ as multi-channel input for the standard independent component analysis algorithm in state space; (**4**) time-delayed state vectors of all estimated independent compounds ***X*** that lie in independent subspaces; (**5**) clustering of the mixing matrix basis that span the independent subspaces of the blind pressure sources and projection of ***X*** in these independent subspaces. Each cluster of state vectors lie in an independent subspace and corresponds to an estimated blind source that contribute to the dynamic in the *BP*_fe_; and (**6**) reconstructed sources in time domain by averaging time-delayed state vectors of the extracted sources and calibration of pressure waveforms with the mean and diastolic blood pressure of *BP*_fe_.

It is generally assumed by classical ICA algorithms that all underlying sources are statistically independent. However, this assumption is not realistic in several biomedical applications. The sources contributing to the underlying dynamic in multi-dimensional signals are not necessarily statistically independent, but some groups of sources lie in statistically independent multi-dimensional subspaces with some dependency within a subspace. In addition, the classical ICA model requires more recordings than underlying sources that should be estimated. Since only one recording of the blood pressure waveform at the femoral artery *BP*_fe_ is available in this study, we extend the classical model into SCICA that represents an extreme case of the overcomplete ICA models, which extract more sources than available sensors. As illustrated in Figure 2, we break up the single-channel recording of *BP*_fe_ into a sequence of time-delayed m-dimensional state vectors using the method of delay so that the underlying temporal dynamics in the recorded *BP*_fe_ is captured, and consider these as multi-channel mixing input for the classical ICA (42). We relaxed the statistical independency between the hidden pressure sources to the statistical independency between subspaces of the underlying pressure waves (48). The principle of the non-linear embedding of scalar time series in state space has been detailed previously (41, 49). In this study, the state space of *BP*_fe_ was reconstructed using the method of delay that was first introduced by Takens (41) and adapted to pulse pressure in our previous work (50). Briefly, the basic idea consists of viewing the signal in a high-dimensional Euclidean space and build an embedding matrix ***M*** by simply decomposing the recorded BP into m-dimensional time-delayed and overlapped state vectors as

(4) $$M=\left[\begin{array}{cccc} BP_{t} & BP_{t+\tau } & \ldots & BP_{t+N\tau }\\ BP_{t+\tau } & BP_{t+2\tau } & \ldots & BP_{t+(N+1)\tau }\\ \vdots & \vdots & \ddots & \vdots \\ BP_{t+(m-1)\tau } & BP_{t+m\tau } & \ldots & BP_{t+(m+N-1)\tau } \end{array}\right]$$

where *τ* is the time lag and *N* is the number of the consecutive state vectors. Takens showed that the Euclidean embedding dimension *m* should be at least as large as the freedom degree, but in real-world applications, the embedding dimension should be sufficiently larger than the Euclidean embedding dimension to account for the inherent noise and the dependencies in the time series data. In practice, *m* can be chosen based on the sampling frequency *f*_s_, the lowest frequency of interest *f*_L_, and the time lag that can be set to 1, i.e.,

(5) $$m\geq \frac{\;f_{s}}{\;f_{L}}\;$$

Once the embedding parameters *m*, *τ*, and *N* are adequately chosen, the embedding matrix ***M*** is rich in information about the underlying temporal dynamics in the recorded pressure wave. In this contribution, *τ* was set to one sampling time corresponding to 2 ms and *m* was set at least to 500 that corresponds to one cardiac cycle (∼1 s) in order to capture the cardiac content of the BP because the lowest frequency of interest of the underlying blood pressure sources is unknown *a priori* (25).

In a pre-processing step, the state vectors of *BP*_fe_ were centered and whitened prior to the ICA separation. The time-delayed state vectors of all blind sources were extracted by ICA using the Fast-ICA algorithm because of its speed and easy implementation (51, 52). It uses the fixed-point scheme for finding the local extrema of the kurtosis

(6) $$Kurt(x)=E\{x^{4}\}-3(E\{x^{2}\}{)}^{2}$$

and solves the blind source problem by maximizing the non-Gaussianity of the sources.

The extracted state vectors with ICA corresponding to single pressure waveforms *BP*_fe,Si_ (*i *= 1, …, *k*) are still correlated compounds. Thus, a post-processing step is needed to group these compounds together. In this step, we cluster the basic functions spanning the independent subspace of estimated pressure waveforms using the K-means algorithm and project the ICA outputs ***X*** into these subspaces. This is done by

(7) $$M^{i}=A(:,C_{i}).W(C_{i},:).X$$

where ***M****^i^* is the matrix of delay and *C_i_* is the cluster of the *i*th extracted pressure wave *BP*_fe,Si_, respectively. In the last stage, the extracted pressure waveforms *BP*_fe,Si_ were reconstructed in measurement space by performing an average of the rows of the their corresponding embedding matrix ***M****^i^*

(8) $$BP_{\mathrm{fe},\mathrm{Si}}(t)=\frac{1}{m}\overset{m}{\underset{k=1}{\sum }}M_{k,(t+k-1)}^{i}\;\;\mathrm{for}\;\;t\;=\;1,2,\ldots ,N$$

The estimated pressure waves with ICA are generally mean free and scaled versions of the original underlying pressure waveforms. To correct this scaling issue, we calibrated the estimated pressure waveforms with *MAP* and *DBP* of the measured intra-arterial *BP*_fe_. We base this procedure on the assumption that the *MAP* and *DBP* are nearly constant in the arterial system.

#### 2.2.2. Evaluation of the SCICA model performance

The accuracy of the SCICA model to estimate the whole c*BP* waveform morphology was evaluated by different performance metrics. The association between the estimated and observed c*BP* waveforms was evaluated by Pearson's correlation. The inter-rater and intra-rater reliability was performed by ICC according to the Shrout and Fleiss schema (43) using the correlation between the estimated and observed c*BP* in two raters. The estimation error was assessed by the mean absolute error (MAE), the root mean square error (RMSE), and the relative RMSE (RRMSE). The MAE is the average error obtained from differences between the recorded and the estimated values of the c*BP*. The RMSE is the average square difference between the observed measured and the predicted values of the c*BP*, in order to obtain a higher weight for large errors. MAE and RMSE are calculated as the following equations:

(9) $$MAE=\frac{1}{n}\overset{n}{\underset{i=1}{\sum }}BP_{\mathrm{ao},i}-BP_{\mathrm{fe},S1_{i}}$$

(10) $$RMSE=\sqrt{\frac{1}{n}\overset{n}{\underset{i=1}{\sum }}{\left(BP_{\mathrm{ao},i}-BP_{\mathrm{fe},S1_{i}}\right)}^{2}}$$

where *n* is the sample size, *BP*_ao,i_ is the *i*th value of the recorded *BP_ao_* at the ascending aorta, and $BP_{\mathrm{fe},S1_{i}}$ is the *i*th value of the estimated *BP*_fe,S1_ with SCICA.

For a parametric test of the SCICA performance to estimate the c*BP* waveform morphology, the RRMSE*_m_* and RRMSE*_r_* were calculated as normalization of the RMSE to the mean of the measured *BP*_ao_, and to the root mean square (RMS) of the *BP*_ao_ as the following equations:

(11) $$RRMSE_{m}=\frac{\sqrt{\frac{1}{n}\sum_{i=1}^{n}{\left(BP_{\mathrm{ao},i}-BP_{\mathrm{fe},S1_{i}}\right)}^{2}}}{\mathrm{mean}(BP_{\mathrm{ao}})}\;.\;100$$

(12) $$RRMSE_{r}=\frac{\sqrt{\frac{1}{n}\sum_{i=1}^{n}{\left(BP_{\mathrm{ao},i}-BP_{\mathrm{fe},S1_{i}}\right)}^{2}}}{\mathrm{RMS}(BP_{\mathrm{ao}})}\;.\;100$$

#### 2.2.3. Estimation of central indices by SCICA

Several pulse pressure parameters are affected by pulse wave transmission and reflection between aorta and peripheral arteries. Thus, we assess additionally the accuracy of the SCICA tool to estimate central indices. The *SBP*, *DBP*, *MAP*, and *PP* were derived from the estimated c*BP* from intra-arterial *BP*_fe_ by SCICA and the intra-aortic *BP*_ao_, and the regression analysis was performed to assess the linear relationship between the estimated and the intra-aortic parameters. The linear fit was quantified by the coefficient of determination *R*^2^, where *R* is the Pearson correlation coefficient. In addition, Bland–Altman plots were used to demonstrate the agreement between estimated and reference parameters. Bland–Altman plots are scatter plots of the mean difference $\;\;\bar{\;\;d}$ against the mean of the two assay measurements. The statistical agreement limits are calculated using the mean difference $\;\bar{\;\;d}$ and its standard deviation *SD* as $\;\;\bar{\;\;d}-1.96\;SD$ and $\;\;\bar{\;\;d}+1.96\;SD$. For good agreement between the two methods, 95% of the differences data points should lie within these limits of agreement.

## 3. Results

### 3.1. Participant characteristics

The pressure recordings were acquired from eight participants with a mean age of 34 ± 11.56 years, predominantly being male (87.5%). Demographic information of the study population is outlined in Table 1.

### 3.2. Estimation of the central aortic pressure by SCICA

**Figure 3:**
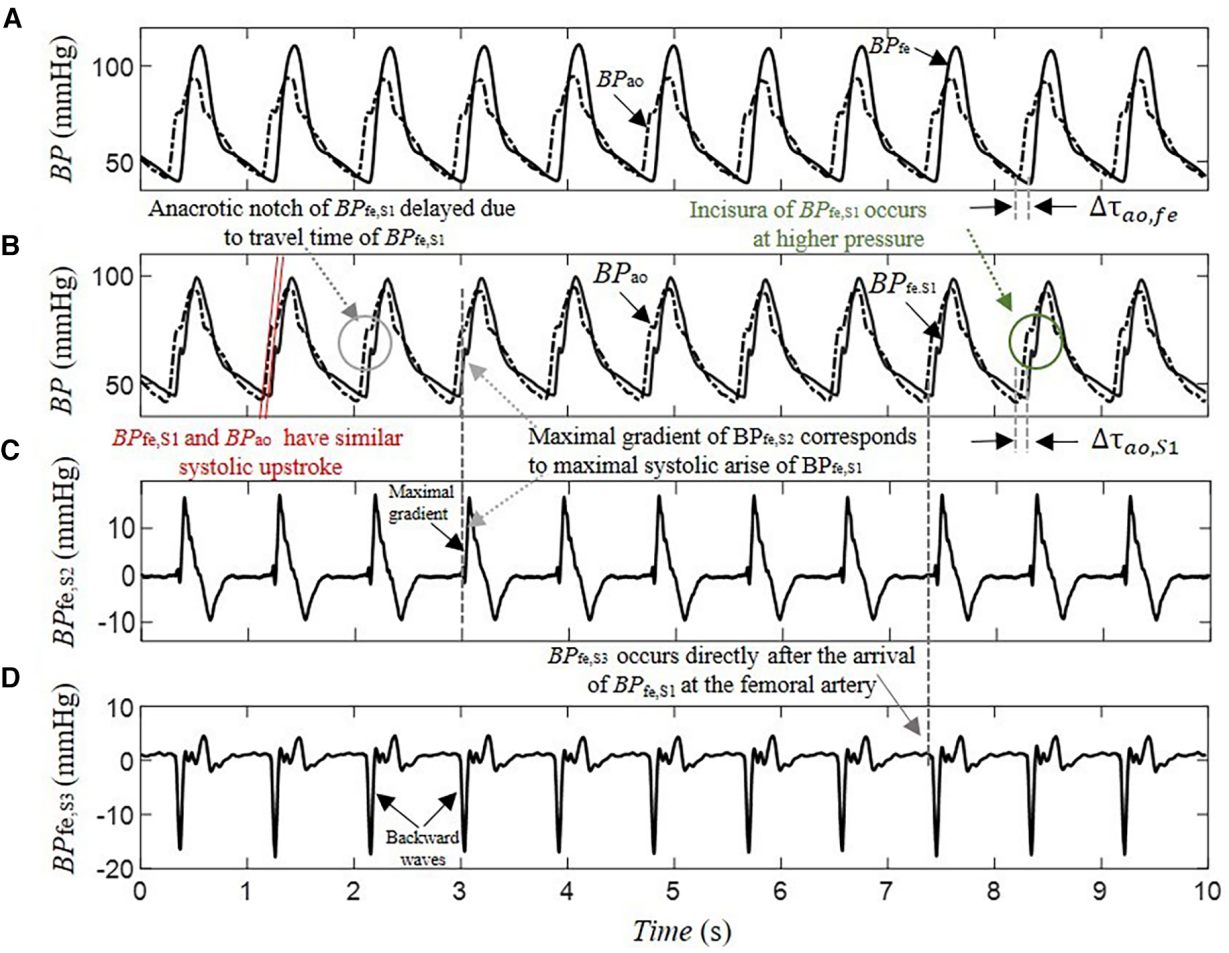
Decomposition of a 10-s segment intra-arterial femoral blood pressure *BP*_fe_ into blind pressure signals contributing to its dynamic using SCICA: (**A**) the recorded intra-arterial *BP*_fe_ (solid line) and the blood pressure at the ascending aorta *BP*_ao_ (dashed line) during coronary artery surgery; (**B**) the first estimated blood pressure *BP*_fe,S1_ (solid line) has the largest contribution to *BP*_fe_ and shows nearly similar time and morphological characteristics to the measured intra-arterial *BP*_ao_ including systolic and diastolic blood pressure, inflection points such as anacrotic notch, systolic rise, and exponential fall-off during the diastole. The anacrotic notch is obvious in the estimated *BP*_fe,S1_ but delayed due to pulse travel time from root to the periphery. However, the incisura of *BP*_fe,S1_ is smother compared to the incisura of the intra-arterial *BP*_ao_. The time delay $\Delta \tau_{\mathrm{ao},\mathrm{fe}\;}$ between *BP*_ao_ and *BP*_fe,S1_ is nearly similar to the time delay $\Delta \tau_{\mathrm{ao},S1}\;$ between *BP*_ao_ and *BP*_fe,S1_; (**C**) the second estimated *BP*_fe,S2_ has similarities with the biphasic blood flow waveform that corresponds to typical blood flow waveform at the femoral artery. It consists of a sharp systolic forward up rise and fall, and a backward wave identical to the reverse flow during early diastole; (**D**) the third estimated *BP*_fe,S3_ has the minimal contribution to the *BP*_fe_ but play an important role in its time arrival at the femoral artery. It is predominantly composed from a backward wave that has inversed, scaled, and compressed form of *BP*_fe,S1_ and occurs directly after the arrival of *BP*_fe,S1_ at the femoral artery. This may indicate that *BP*_fe,S3_ corresponds to reflected waves of *BP*_fe,S1_.

**Figure 4:**
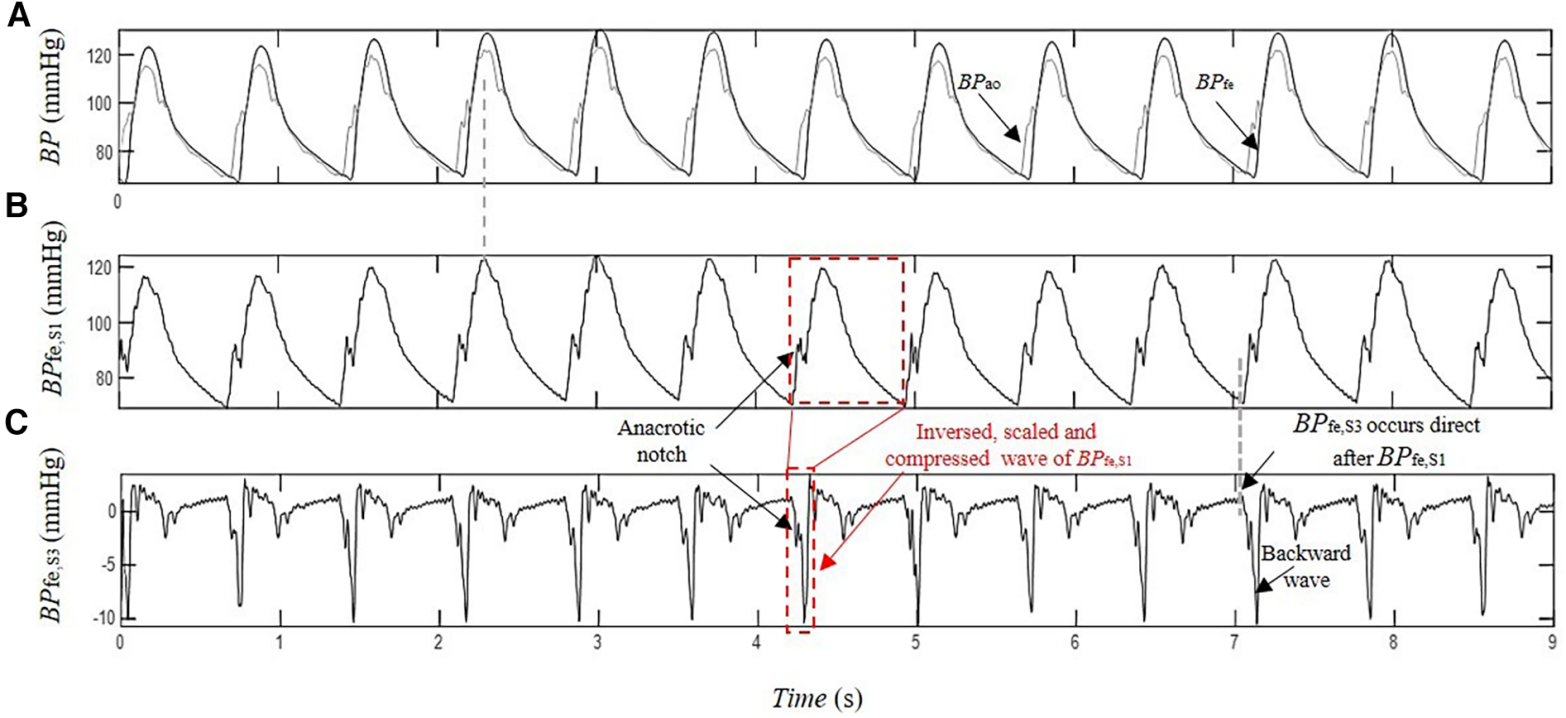
Estimation of the contribution of the pressure wave reflection by SCICA: (**A**) recorded intra-arterial blood pressure at the femoral artery *BP*_fe_ (black) and blood pressure at the ascending aorta *BP*_ao_ (gray) during coronary artery surgery; (**B**) first estimated pressure waveform *BP*_fe,S1_ with similar morphological and time characteristics such as *BP*_ao_; and (**C**) the third extracted pressure waveform *BP*_fe,S3_ has the minimal contribution to the recorded *BP*_fe_. The first backward wavelet of *BP*_fe,S3_ occurs directly after the arrival of *BP*_fe,S1_ at the femoral artery. It has an inversed, scaled, and compressed form of the *BP*_fe,S1_ that may correspond to a local reflection of *BP*_fe,S1_ at the femoral artery. The other wavelets contributing to *BP*_fe,S3_ morphology occur in the systole and early diastole The late diastolic part of *BP*_fe,S3_ is nearly wave free.

**Figure 5:**
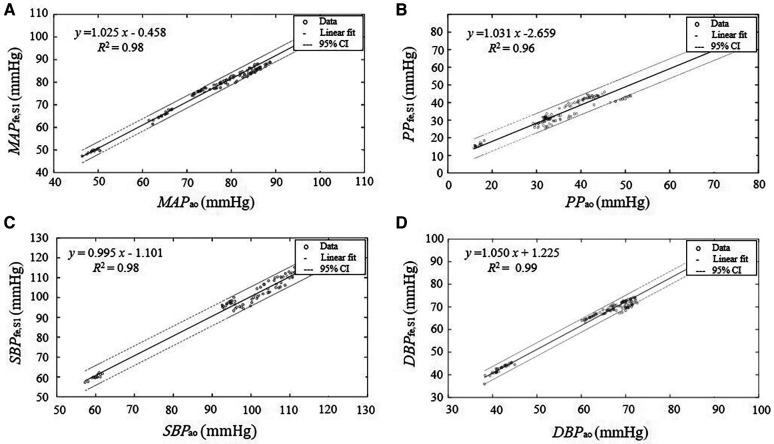
Linear regression between the parameters derived from the estimated central pressure *BP*_fe,S1_ from the pressure recording at the femoral artery *BP*_fe_ by SCICA and the central parameters derived from the intra-aortic pressure *BP*_ao_. The solid lines represent the regression lines of best fit and the dashed lines represent 95% confidence intervals (95% CI) for the regression lines. The regression analysis shows a strong linear relationship between the estimated central indices by SCICA and intra-aortic indices: (**A**) *MAP*_fe,S1_ and *MAP*_ao_, (**B**) *SBP*_fe,S1_ and S*BP*_ao_, (**C**) *DBP*_fe,S1_ and *DBP*_ao_, and (**D**) *PP*_fe,S1_ and *PP*_ao_ with very high coefficients of determination *R*^2^ of 0.98, 0.98, 0.99, and 0.96, respectively.

The separation of *BP*_fe_ into their underlying blind sources by SCICA showed in all enrolled patients in this study a presence of three pressure waveform sources contributing to determination of its morphology. As depicted in Figure 3, the first estimated component *BP*_fe,S1_ shows the largest contribution to *BP*_fe_. The second extracted source *BP*_fe,S2_ is similar to the biphasic blood waveform of the typical blood flow at the femoral artery and plays a pivotal role in the augmentation of pressure in the systole. It consists of a sharp systolic forward up rise and fall, and a backward wave identical to the reverse flow during early diastole. The third component *BP*_fe,S3_ provides the smallest contribution to *BP*_fe_, but plays an important role in the time arrival of the *BP*_fe_. The *BP*_fe,S3_ is predominantly composed of a backward wave that demonstrates an inverted, scaled, and compressed form of *BP*_fe,S1_ and occurs directly after the arrival of *BP*_fe,S1_ at the femoral artery, as depicted in Figures 3D, 4C. This indicates that this backward wave probably represents a reflection of *BP*_fe,S1_ that arises locally at the femoral artery, and that *BP*_fe,S3_ may represent an overlapping of discrete reflections of the incident *BP*_fe,S1_ occurring between aorta and femoral arteries because of impedance discontinuities due to radius and tonus changes. The inflection point corresponding to dicrotic notch occurred at a relatively higher pressure as the incisura of the measured c*BP*, as illustrated in Figures 4B, 5B, and was not obvious in the reflected wave in all patients, as shown in Figure 3D compared to Figure 4C.

*BP*_fe,S1_ and *BP*_ao_ arise with similar systolic upstroke, as shown in Figure 3B. After closure of the aortic valve, *BP*_ao_ and *BP*_S1_,_fe_ decrease to the baseline levels at the end diastole with nearly similar exponential diastolic decay. The time delay Δ*τ*_ao,S1_ between *BP*_fe,S1_ and *BP*_ao_ is close to the time delay Δ*τ*_ao,fe_ between the measured *BP*_fe_ and *BP*_ao_ that corresponds to the transit time of the pressure wave from the ascending aorta to the femoral artery. Moreover, the pressure wave activity of *BP*_fe,S2_ and *BP*_fe,S3_ during diastole was minimal and very close to zero, matching the diastolic flow. *BP*_fe,S1_ was the main driver of the exponential pressure fall-off in diastole in all patient recordings.

Furthermore, the performance metrics *MAE, RMSE, RRMSE_m_*, and *RRMSE_r_* evaluating the difference between the estimated *BP*_fe,S1_ and the measured *BP*_ao_ using a window duration of 10 s and a time lag of 2 ms were relatively consistent across all subjects with mean values 0.159 ± 1.629 mmHg, 5.153 ± 0.957 mmHg, 5.424% ± 1.304%, and 5.354% ± 1.263% respectively, as shown in Table 2. However, with decreasing duration of *BP*_fe_ (but no shorter than one cardiac cycle), the difference between the estimated and measured c*BP* morphologies increased relatively. However, the performance of the SCICA model was sufficiently good with *RMSE* ≤10 mmHg, *RRMSE_m_* ≤10%, and *RRMSE_r_* ≤10%. In addition, the high ICC of 0.94 showed a good inter-subject and intra-subject consistency of the *cBP* estimation by SCICA approach. Furthermore, the high number of state vectors due to low embedding dimension led to distorted pressure waveform, smoothed, or suppressed notch points. The embedding dimension was the most sensitive embedding parameter that showed strong intra-subject and inter-subject variability.

**Table 2:** Performance evaluation of SCICA approach to estimate the whole central blood pressure waveform morphology from the femoral artery.

Patient	*MAE* (mmHg)	*RMSE* (mmHg)	*RRMSE_m_* (%)	*RRMSE_r_* (%)	*R*_1_	*R*_2_
#1	0.537	5.182	5.709	5.665	0.961	0.960
#2	1.238	6.309	5.847	5.810	0.951	0.981
#3	−1.158	4.207	4.780	4.713	0.984	0.984
#4	0.663	6.348	6.945	6.684	0.965	0.957
#5	1.853	4.849	4.376	4.354	0.978	0.982
#6	−0.916	3.607	3.627	3.578	0.974	0.973
#7	−2.761	5.657	7.432	7.367	0.890	0.858
#8	1.812	5.061	4.675	4.659	0.940	0.953
Total	0.159 ± 1.629	5.153 ± 0.957	5.424 ± 1.304	5.354 ± 1.263	0.955 ± 0.301	0.956 ± 0.415

*RRMSE_m_*, relative RMSE normalized with the mean of the observed c*BP*; *RRMSE*_r_ relative RMSE normalized with the root mean square of the observed c*BP*; *R*_1_ and *R*_2_, correlation between the estimated and measured c*BP* in first and second measurement (rater), respectively.

The metrics were calculated using *BP*_fe_ waves with a duration of 10 s, and a constant time lag of *τ *= 2 ms for all patients.

### 3.3. Evaluation of derived parameters from estimated aortic blood pressure by SCICA

In this part, we investigate the accuracy of SCICA to estimate central indices from *BP*_fe,S1_. As depicted in Figure 5, the regression analysis showed a strong linear fit between the estimated parameters MAP_fe,S1_, *SBP*_fe,S1_, *DBP*_fe,S1_, and *PP*_fe,S1_ derived from the extracted *BP*_fe,S1_ and the intra-aortic parameters MAP_ao_, *SBP*_ao_, *DBP*_ao_, and *PP*_ao_ with very high coefficient of determination *R*^2^ of 0.9821, 0,9811, 0.9910, and 0.9611 respectively, and following regression functions:

(13) $$MAP_{\mathrm{fe},S1}=1.025\;MAP_{\mathrm{ao}}-0.458$$

(14) $$SBP_{\mathrm{fe},S1\;}=0.995\;\;SBP_{\mathrm{ao}}-1.101$$

(15) $$DBP_{\mathrm{fe},S1}=1.050\;\;DBP_{\mathrm{ao}}+1.225\;$$

(16) $$PP_{\mathrm{fe},S1}=1.031\;PP_{\mathrm{ao}}-2.659$$

**Figure 6:**
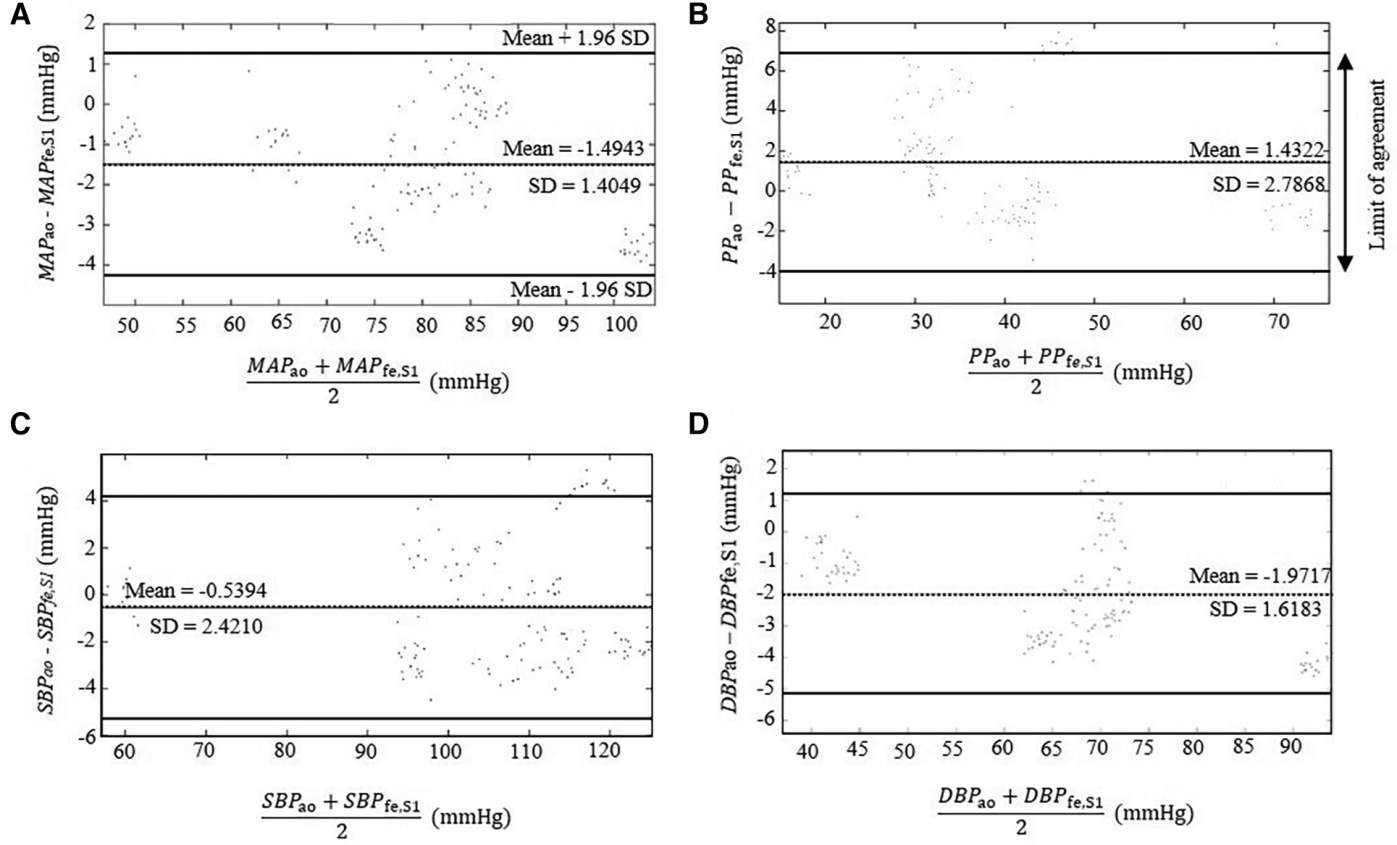
Bland–Altman plots comparing the estimated parameters derived from the extracted *BP*_fe,S1_ from the intra-artery *BP*_fe_ using SCICA with the intra-aortic parameters derived from intra-aortic *BP*_ao_: (**A**) *MAP*, (**B**) *PP*, (**C**) *SBP*, and (**D**) *DBP.* The dotted lines show the mean difference between the reference and estimated measurements. The 95% limits of agreement are represented with bold lines and are computed as the mean difference plus or minus 1.96 times of its standard deviation *SD*: mean − 1.96 *SD* and mean + 1.96 *SD*. Bland–Altman plots show a good agreement between the estimated central parameters by SCICA and the intra-aortic features because 95% of mean differences points lie within the limit of agreements.

As illustrated in Figure 6 and summarized in Table 3, Bland–Altman plots show good agreements between estimated and intra-aortic features with a mean difference of −0.54 ± 2.42 mmHg [95% confidence interval (CI): −5.28 to 4.21] for *SBP*, −1.97 ± 1.62 mmHg (95% CI: −5.14 to 1.20) for *DBP*, −1.49 ± 1.40 mmHg (95% CI: −4.25 to 1.26) for *MAP*, and 1.43 ± 2.79 (95% CI: −4.03 to 6.89) for *PP*. Most data points (more than 95%) lie within the limits of agreements −0.54 ± 4.74 mmHg, −1.97 ± 3.18 mmHg, −1.49 ± 2.74 mmHg, and 1.43 ± 5.47 mmHg for *SBP*, *DBP*, *MAP*, and *PP*, respectively.

**Table 3:** Evaluation of the SCICA approach to estimate the central parameters using the regression and Bland–Altman analyses.

Biomarkers	Mean difference^a^	*SD* of mean^b^	95% CI	*R* ^2^
SBP (mmHg)	−0.54	2.42	−5.28 to 4.20	0.98
DBP (mmHg)	−1.97	1.62	−5.14 to 1.20	0.99
MAP (mmHg)	−1.49	1.40	−4.25 to 1.26	0.98
PP (mmHg)	1.43	2.79	−4.03 to 6.89	0.96

CI, confidence interval.

^a^ Mean difference between the intra-aortic and estimated parameters.

^b^ Standard deviation of the mean difference.

According to the high morphological association between *BP*_ao_ and *BP*_fe,S1_ with an averaged high correlation of 0.96, and a high ICC of 0.94, as well as the relative low morphological difference in-between with *MAE* and *RMSE* lower than 6 mmHg, *RRMSE* lower than 6%, and the accurate estimation of the central parameters including *SBP*, *DBP*, *PP*, and *MAP,* the estimated pressure wave *BP*_fe,S1_ was considered to be an estimate of the c*BP*.

## 4. Discussion

We introduced in this study a novel cardiovascular tool for the separation of the multivariate *pBP* into their blind pressure waveforms in humans without a priory assumption on the arterial model or the origins of these pressure compounds. To our knowledge, the proposed SCICA approach is the first ICA tool that estimates the *cBP* waveform using only one recording of intra-arterial *BP*_fe_. Most ICA algorithms applied to the decomposition of the BP rely on the spatial analysis, i.e., use a synchronized multi-recording of BP at distal arteries to estimate the *cBP*. The proposed SCICA approach in this contribution can extract multiple underlying pressure sources using only the temporal information inherent within a single recording of *pBP* using a non-linear embedding in a high-dimensional state space.

SCICA estimated three independent pressure waveforms from *BP*_fe_ that interact linearly with each other to determine the pressure wave morphology and provided an explanation of shape distortion at this artery. Similar results were observed in finger PPG decomposition by SCICA in healthy subjects in our previous works (50, 53), in radial pressure waveform in our recent ongoing works, and can be validated in other distal and proximal arteries. The first extracted pressure wave *BP*_fe,S1_ showed in all patient recordings temporal and morphological characteristic relatively similar to intra-aortic *BP*_ao_ at the ascending aorta and played the dominant role in determining the morphology of the pressure waveform at the femoral artery. According to the relatively consistent *MAE* and *RMSE* (<6 mmHg) across the study subjects, as well as the very good *RRMSE* lower than 6%, the *BP*_fe,S1_ could be considered a good estimate of the c*BP* waveform. The difference between the estimated and measured c*BP* waveforms increases with decreased duration of the peripheral blood pressure and the performance decreases, but not considerably. In the literature, there are no enough specific guidelines for an optimal selection of duration, m or *τ*; only general recommendations are available (54). Our preliminary results indicated that the shorter duration of the *pBP* (but not shorter than one cardiac cycle) is sufficient for a relatively good performance with *RMSE* ≤10 and *RRMSE* ≤10%. This may be due to the fact that the important cardiac patterns in our study are already apparent at short lengths such as one cardiac cycle. Furthermore, the most available methods and commercial devices that estimate non-invasively the *cBP* from the *pBP* evaluate mainly the performance of their estimation methods according to the error between the estimated and observed central parameters such as *SBP, DBP, MAP, PP*, or augmentation index (13, 15, 55, 56). The literature is quite sparse considering the accuracy of the non-invasive estimation of the whole *cBP* morphology from *pBP*. Several blind source separation approaches and machine learning models reconstructed the *cBP* from multiple measurements of *pBP*. However, few information about the accuracy of the whole waveform reconstruction is available. Magbool et al. reported an average RMSE lower than 7 mmHg using different machine learning models, and an average *RMSE* lower than 4 mmHg using hybrid machine learning and multi-channel blind source separation identification models. However, virtual databases were used to train the models (29).

The morphological difference between the estimated c*BP* with SCICA approach and the recorded c*BP* waveform at the ascending aorta could be in part due to the technical issues in clinical settings that cause a damping of *pBP* waveforms. On the other hand, the SCICA approach estimates the *cBP* as a blind source without additional pressure compounds contrary to the measured c*BP* at the ascending aorta that represent a summation of the forward pressure wave and other pressure waves such as reflected waveforms occurring at distal and proximal sites. Thus, it is expected that the predicted pressure waveform has some difference in the key points such anacrotic notch and incisura. This may explain the occurrence of the incisura of the estimated *cBP* with SCICA in higher pressure compared with the measured *BP*_ao_, as previously explained in the introduction und illustrated in Figure 1. Moreover, our results showed that a small embedding dimension led to smoothed notches.

The second estimated blind source *BP*_fe,S2_ showed a similarity with a biphasic blood flow waveform that corresponds to typical flow wave at the femoral artery in all patients and seems to play a pivotal role in the augmentation and morphological change during the systole at this artery. Similar morphology showed *BP*_exc_ in previous works using the decomposition of *cBP* and *pBP* by the RWC model that assumes that the *cBP* and *pBP* are summation of a reservoir pressure that accounts for global arterial compliance of the arteries and a *BP*_exc_ that is determined by local arterial characteristics (57). The *BP*_res_ is homogeneous throughout the arterial system, but varies temporally with a time lag that depends on the travel time from the root to the distal location and properties of the arteries (39) and could be estimated at any peripheral artery. Accumulating evidence suggested that *BP*_exc_ at the aorta and peripheral arteries correlate directly with the local blood flow waveform (39, 40). Moreover, the *BP*_fe,S3_ showed the smallest contribution to the *BP*_fe_ and consisted predominantly of a backward pressure wave that may represent wave reflection of *BP*_fe,S1_. This agrees with the less prominent role of the wave reflection in pressure augmentation. Mounting evidence suggests that discrete wave reflection plays a less important role in the shape determination of the *cBP* waveform than originally conceived (58–60). Furthermore, the results of SCICA showed that the wave activity in the late diastole is minimal and mainly related to *BP*_fe,S1_, whereas the wave activity of *BP*_fe,S2_ and *BP*_fe,S3_ occurred mainly during the systole. These findings are in accordance with the RWC theory. A growing body of literature supports the largest contribution of *BP*_res_ to the pressure wave during diastole (57).

Adding to this, SCICA estimated the central parameters with a clinically acceptable accuracy. The Bland–Altman analysis showed a very good agreement between intra-aortic and the estimated parameters from the femoral artery with SCICA. The mean errors of all estimated central parameters were generally less than 3 mmHg with an acceptable scatter of the data points in a confidence interval between −5 and 6 mmHg. Of particular significance was the estimated *cSBP* with a mean error of –0.54 mmHg and a precision (SD) of 2.421 mmHg (95% CI: −5.284 to 4.205), which both, mean error and *SD*, fall within the limits proposed by the guidelines of the Association for the Advancement of Medical Instrumentation, namely, a mean error lower than 5 mmHg and a precision lower than 8 mmHg. In addition, the estimated *cSBP* with SCICA in this study was very similar to the value of the mean error reported in systematic reviews and meta-analysis of non-invasive *cBP* validation studies of commercial devices. Cheng et al. reported a small error of estimated *cSBP* with a mean and standard deviation of difference −1.1 ± 4.1 mmHg (95% CI: −9.1 to 6.9 mmHg) (12). In a second meta-analysis, the mean error of estimate c*SBP* in devices that was calibrated using invasively recorded BP was −1.08 mmHg (95% CI: −2.81 to 0.65 mmHg) (56).

Of particular importance regarding the SCICA tool is the determination of the pressure waveforms underlying the pressure dynamics and the time delay in-between. These data hide valuable information about the augmentation of pressure, reflection site of the pressure wave in the circulatory system, and state of the endothelium, and could be used to derive new augmentation and reflection indices. Compared with methods currently used, there is no need for any supplementary device to calibrate the extracted pressure waveforms or a plurality of recording to conceptualize the underlying arterial hemodynamic. Only the statistical independency of the extracted blind source pressures, *MAP*, and *DBP* of the measured intra-arterial *BP*_fe_ was used to calibrate the estimated pressure waves. Most of the commercial devices consider the widely accepted assumption that the *MAP* and *DBP* are nearly constant in the arterial tree to calibrate *pBP*. No additional brachial cuff calibration was required in this study. This may indicate that the newly developed SCICA method could avoid additional errors induced with the not accurate brachial cuff BP measurement. Different validation studies reported that the discrepancy between estimated *SBP* by arterial tonometer and intra-aortic *SBP* appears to be largely related to the calibration process of non-invasively assessed pulse pressure waveform. Moreover, in contrary to the commercially available device for generating *cBP* non-invasively from the peripheral arteries that use a generalized transfer function, the SCICA tool is time- and subject-specific. No averaging over time or over a population is required.

Despite the aforementioned findings, our study has several limitations. The main limitation of this study is the validation in a small data set with a prevalence of male participants. Second, the pressure recordings were originally acquired in patients undergoing cardiac catheterization, who are not necessarily representative of the general population. Nonetheless, similar pressure waveform estimation was observed in PPG decomposition with SCICA in our previous works (50, 53) and in radial BP in healthy subjects. We believe that the results can be extrapolated to the general population with some confidence. A third limitation is the use of only one ICA algorithm, the Fast-ICA algorithm that assumes that the recorded BP signal is a linear combination of the blind pressure waves contributing to its dynamics. This is, however, not necessarily the case. We cannot rule out that more sources are available and could be convolutively added in the detected waveforms. Thus, non-linear and convolutive ICA algorithms should be investigated in future works to compare the accuracy of the ICA approaches and the number of pressure waveforms. Fourth, the embedding parameters play a decisive role in the detection of the underlying dynamics and estimation of the underlying pressure compounds. In the present contribution, only the time of delay embedding method was implemented. This method has some limitations in the presence of noise (61). Thus, in a future investigation, several embedding approaches such as derivative embedding or other dynamical prediction methods should be implemented and optimized in a larger population to analyze the impact of the embedding parameters for state space on the performance of the SCICA approach. In the present research, the pressure waveforms were sampled with a constant sampling frequency of 500 Hz in clinical settings. This corresponds to a time lag of 2 ms that provided a good model performance. Generally, the sampling frequency plays a key role in the determination of the embedding time lag. A high sampling rate led to redundant information due to the small time lag, and similarity of the consecutive samples (the trajectory in state space is close to the diagonal), whereas a low sampling rate led to a large embedding time lag that may cause a distortion of the underlying dynamic (the geometric structure of the trajectory in state space become more complex and the dispersed) (61). Thus, the impact of different sampling rates on the performance of the SCICA model should be investigated in future works. In addition, the embedding dimension was the most sensitive embedding parameter that showed inter-subject and intra-subject variability and should be analyzed in a larger population using different methods. Finally, the proposed SCICA model has similarities with the reservoir-excess arterial model. Thus, a comparison of the extracted pressure compounds with both models should be compared in future investigation in order to explain the physiology of the estimated pressure waves.

## Data Availability

The original contributions presented in the study are included in the article/Supplementary Material. Further inquiries can be directed to the corresponding author, as well as author EW (Ernst.wellnhofer@charite.de) and Prof. Goubergrits (leonid.goubergrits@charite.de).

## References

[1] Wang K Cheng H Chuang S Spurgeon HA. Central or peripheral systolic or pulse pressure: which best relates to target-organs and future. J Hypertens. (2011) 27(201):461–7. 10.1097/hjh.0b013e3283220ea4 PMC3178100 19330899

[2] Nelson MR Stepanek J Cevette M Covalciuc M Hurst RT Tajik AJ. Noninvasive measurement of central vascular pressures with arterial tonometry: clinical revival of the pulse pressure waveform? Mayo Clin Proc. (2010) 85(5):460–72. 10.4065/mcp.2009.0336 20435839 PMC2861976

[3] Kaniusas E. Biomed signals and sensors I. Springer Heidelberg Dordrecht London New York. (2012). p. 1–21. 10.1007/978-3-642-24843-6

[4] Wood EH Kroeker EJ. Comparison of simultaneously recorded central and peripheral arterial pressure pulses during rest, exercise and tilted position in man. Circ Res. (1955) 3(6):623–32. 10.1161/01.RES.3.6.623 13270378

[5] Gravlee GP Wong AB Adkins TG Douglas Case L Pauca AL. A comparison of radial, brachial, and aortic pressures after cardiopulmonary bypass. J Cardiothorac Anesth. (1989) 3(1):20–6. 10.1016/0888-6296(89)90006-9 2520634

[6] O’Rourke MF Adji A. Noninvasive studies of central aortic pressure. Curr Hypertens Rep. (2012) 14(1):8–20. 10.1007/s11906-011-0236-5 22083214

[7] Asmar RG London GM O’Rourke ME Safar ME. Improvement in blood pressure, arterial stiffness and wave reflections with a very-low-dose perindopril/indapamide combination in hypertensive patient: a comparison with atenolol. Hypertension. (2001) 38(4):922–6. 10.1161/hy1001.095774 11641310

[8] Morgan T Lauri J Bertram D Anderson A. Effect of different antihypertensive drug classes on central aortic pressure. Am J Hypertens. (2004) 17(2):118–23. 10.1016/j.amjhyper.2003.09.012 14751652

[9] Mceniery CM Cockcroft JR Roman MJ Franklin SS Wilkinson IB. Central blood pressure: current evidence and clinical importance. Eur Heart J. (2014) 35(26):1719–25. 10.1093/eurheartj/eht565 24459197 PMC4155427

[10] Pauca AL O’Rourke MF Kon ND. Prospective evaluation of a method for estimating ascending aortic pressure from the radial artery pressure waveform. Hypertension. (2001) 38(4):932–7. 10.1161/hy1001.096106 11641312

[11] Segers P Mahieu D Kips J Van Bortel LM. The use of a generalized transfer function: different processing, different results!. J Hypertens. (2007) 25(9):1783–7. 10.1097/HJH.0b013e3282ef5c5f 17762638

[12] Cheng HM Lang D Tufanaru C Pearson A. Measurement accuracy of non-invasively obtained central blood pressure by applanation tonometry: a systematic review and meta-analysis. Int J Cardiol. (2013) 167(5):1867–76. 10.1016/j.ijcard.2012.04.155 22622052

[13] Gao M Rose WC Fetics B Kass DA Chen CH Mukkamala R. A simple adaptive transfer function for deriving the central blood pressure waveform from a radial blood pressure waveform. Sci Rep. (2016) 6(September):1–9. 10.1038/srep33230 27624389 PMC5021949

[14] Pauca AL Kon ND O’Rourke MF. The second peak of the radial artery pressure wave represents aortic systolic pressure in hypertensive and elderly patients. Br J Anaesth. (2004) 92(5):651–7. 10.1093/bja/aeh121 15003985

[15] Chemla D Millasseau S Hamzaoui O Teboul J-L Monnet X Michard F New method to estimate central systolic blood pressure from peripheral pressure: a proof of concept and validation study. Front Cardiovasc Med. (2021) 8(December):1–10. 10.3389/fcvm.2021.772613 PMC8714848 34977186

[16] Schultz MG Picone DS Armstrong MK Black JA Dwyer N Roberts-Thomson P Validation study to determine the accuracy of central blood pressure measurement using the Sphygmocor Xcel cuff device. Hypertension. (2020) 76:244–50. 10.1161/HYPERTENSIONAHA.120.14916 32475318

[17] Sharman JE Lim R Qasem AM Coombes JS Burgess MI Franco J Validation of a generalized transfer function to noninvasively derive central blood pressure during exercise. Hypertension. (2006) 47(6):1203–8. 10.1161/01.HYP.0000223013.60612.72 16651459

[18] Williams B Lacy PS Yan P Hwee CN Liang C Ting CM. Development and validation of a novel method to derive central aortic systolic pressure from the radial pressure waveform using an n-point moving average method. J Am Coll Cardiol. (2011) 57(8):951–61. 10.1016/j.jacc.2010.09.054 21329842

[19] Hickson SS Butlin M Mir FA Graggaber J Cheriyan J Khan F The accuracy of central SBP determined from the second systolic peak of the peripheral pressure waveform. J Hypertens. (2009) 27(9):1784–8. 10.1097/HJH.0b013e32832e0b58 19702000

[20] Zhang Y Asada HH. *Multi-channel blind system identification for cardiovascular monitoring*. 2. US Patent No 7169111B2 (2007).

[21] Hahn JO Reisner A Asada HH. A blind approach to reconstruction of aortic blood pressure waveform using gray-box identification of multiple pressure transfer channels. Proc Am Control Conf. (2006) 2006:3415–20. 10.1109/ACC.2006.1657246

[22] Hahn JO McCombie DB Reisner AT Hojman HM Harry A. Identification of multichannel cardiovascular dynamics using dual Laguerre basis functions for noninvasive cardiovascular monitoring. IEEE Trans Control Syst Technol. (2010) 18(1):170–6. 10.1109/TCST.2008.2009996

[23] Swamy G Ling Q Li T Mukkamala R. Blind identification of the aortic pressure waveform from multiple peripheral artery pressure waveforms. Am J Physiol Heart Circ Physiol. (2007) 292(5):2257–64. 10.1152/ajpheart.01159.2006 17208992

[24] Calhoun VD Adali T Pearlson GD Pekar JJ. Spatial and temporal independent component analysis of functional MRI data containing a pair of task-related waveforms. Hum Brain Mapp. (2001) 13(1):43–53. 10.1002/hbm.1024 11284046 PMC6871956

[25] James CJ Lowe D. Single channel analysis of electromagnetic brain signals through ICA in a dynamical systems framework. *2001 Conference Proceedings of the 23rd Annual International Conference of the IEEE Engineering in Medicine and Biology Society*. Istanbul, Turkey (2001). pp. 1974–7. 10.1109/IEMBS.2001.1020616

[26] Mourad N Kirubarajan T Reilly JP Hasey G Maccrimmon D. Constrained sparse component analysis with applications to EEG / MEG signal processing. Electr Eng. (2011) 1–25.

[27] James CJ Hesse CW. Independent component analysis for biomedical signals. Physiol Meas. (2005) 26(1):R15–39. 10.1088/0967-3334/26/1/R02 15742873

[28] Varanini M Massoni M Marraccini P KozAkova M Djukic M Bamoshmoosh M. Aorta_neural_network.pdf. Comput Cardiol. (2003) 30:501–4. 10.1109/CIC.2003.1291202

[29] Magbool A Bahloul MA Ballal T Al-Naffouri TY Laleg-Kirati TM. Aortic blood pressure estimation: a hybrid machine-learning and cross-relation approach. Biomed Signal Process Control. (2021) 68(May):102762. 10.1016/j.bspc.2021.102762

[30] Li P Laleg-Kirati TM. Central blood pressure estimation from distal PPG measurement using semiclassical signal analysis features. IEEE Access. (2021) 9:44963–73. 10.1109/ACCESS.2021.3065576

[31] Westerhof N Segers P Westerhof BE. Wave separation, wave intensity, the reservoir-wave concept, and the instantaneous wave-free ratio: presumptions and principles. Hypertension. (2015) 66(1):93–8. 10.1161/HYPERTENSIONAHA.115.05567 26015448

[32] Cameron JD. Wave intensity analysis and central blood pressure. Hypertension. (2009) 54:958–9. 10.1161/HYPERTENSIONAHA.109.137638 19720953

[33] Broyd CJ Nijjer S Sen S Petraco R Jones S Al-Lamee R Estimation of coronary wave intensity analysis using noninvasive techniques and its application to exercise physiology. Am J Physiol Heart Circ Physiol. (2016) 310:H619–27. 10.1152/ajpheart.00575.2015 26683900 PMC4778269

[34] Sugawara M Niki K Ohte N Okada T Harada A. Clinical usefulness of wave intensity analysis. Med Biol Eng Comput. (2009) 47(2):197–206. 10.1007/s11517-008-0388-x 18763005

[35] Armstrong MK Schultz MG Hughes AD Picone DS Sharman JE. Physiological and clinical insights from reservoir-excess pressure analysis. J Hum Hypertens. (2021) 35(9):758–68. 10.1038/s41371-021-00515-6 33750902 PMC7611663

[36] Aizawa K Casanova F Gates PE Mawson DM Gooding KM Strain WD Reservoir-excess pressure parameters independently predict cardiovascular events in individuals with type 2 diabetes. Hypertension. (2021) 78(1):40–50. 10.1161/HYPERTENSIONAHA.121.17001 34058850 PMC7611068

[37] Armstrong MK Schultz MG Hughes AD Picone DS Black JA Dwyer N Excess pressure as an analogue of blood flow velocity. J Hypertens. (2021) 39(3):421–7. 10.1097/HJH.0000000000002662 33031183 PMC7116698

[38] Parker KH Alastruey J Stan GB. Arterial reservoir—excess pressure and ventricular work. Med Biol Eng Comput. (2012) 50(4):419–24. 10.1007/s11517-012-0872-1 22367750

[39] Hughes AD Parker KH. The modified arterial reservoir: an update with consideration of asymptotic pressure (P∞) and zero-flow pressure (Pzf). Proc Inst Mech Eng H. (2020) 234(11):1288–99. 10.1177/0954411920917557 32367773 PMC7705641

[40] Narayan O Parker KH Davies JE Hughes AD Meredith IT Cameron JD. Reservoir pressure analysis of aortic blood pressure: an in-vivo study at five locations in humans. J Hypertens. (2017) 35(10):2025–33. 10.1097/HJH.0000000000001424 28582283 PMC5581543

[41] Deyle ER Sugihara G. Generalized theorems for nonlinear state space reconstruction. PLoS One. (2011) 6(3):e18295. 10.1371/journal.pone.0018295 21483839 PMC3069082

[42] Tharwat A. Independent component analysis: an introduction. Trends Cogn Sci. (2021) 17(2):222–49. 10.1016/j.aci.2018.08.006 15866182

[43] Shrout PE Fleiss JL. Intraclass correlations: uses in assessing rater reliability. Psychol Bull. (1979) 86(2):420–8. 10.1037/0033-2909.86.2.420 18839484

[44] Bonett DG. Sample size requirements for estimating intraclass correlations with desired precision. Stat Med. (2002) 21(9):1331–5. 10.1002/sim.1108 12111881

[45] Walter SD Eliasziw M Donner A. Sample size and optimal designs for reliability studies. Stat Med. (1998) 17(1):101–10. 10.1002/(SICI)1097-0258(19980115)17:1<101::AID-SIM727>3.0.CO;2-E 9463853

[46] Pati R Pujari AK Gahan P Kumar V. Independent component analysis: a review with emphasis on commonly used algorithms and contrast function. Comput y Sist. (2021) 25(1):97–115. 10.13053/cys-25-1-3449

[47] Sheehan MP Davies ME. Compressive independent component analysis: theory and algorithms. Inf Inference A J IMA. (2023) 12(1):551–89. 10.1093/imaiai/iaac016

[48] Póczos B Lorincz A. Independent subspace analysis using k-nearest neighborhood distances. In: Duch W, Kacprzyk J, Oja E, Zadrożny S, editors. Lecture notes in computer science (including *Lecture Notes in Artificial Intelligence and Lecture Notes in Bioinformatics*). Berlin, Heidelberg: Springer (2005) Vol. 3697. p. 163–8.

[49] Matilla-García M Morales I Rodríguez JM Ruiz Marín M. Selection of embedding dimension and delay time in phase space reconstruction via symbolic dynamics. Entropy (Basel). (2021) 23(2):221. 10.3390/e23020221 PMC7916852 33670103

[50] Gbaoui L Kaniusas E. Decomposition of photoplethysmographical arterial pulse waves by independent component analysis: possibilities and limitations. In: Mukhopadhyay SC, Lay-Ekuakille A, editors. Lecture notes in electrical engineering. Berlin, Heidelberg: Springer (2010). p. 166–85. 10.1007/978-3-642-05167-8_11

[51] Hyvärinen A Oja E. A fast fixed-point algorithm for independent component analysis. Neural Comput. (1997) 9(7):1483–92. 10.1162/neco.1997.9.7.1483

[52] Hyv A. Fast and robust fixed-point algorithms for independent component analysis. (1999) 10(3):626–34. 10.1109/72.761722 18252563

[53] Gbaoui L Kaniusas E. Arterial pulse wave decomposition by independent component analysis. *2009 IEEE International Workshop on Medical Measurements and Applications, MeMeA*. 29-30 May 2009. Cetraro, Italy: IEEE (2009). p. 111–5.

[54] Cuesta-Frau D Murillo-Escobar JP Orrego DA Delgado-Trejos E. Embedded dimension and time series length. Practical influence on permutation entropy and its applications. Entropy. (2019) 21(4):1–25. 10.3390/e21040385 PMC7514869 33267099

[55] Chemla D Agnoletti D Jozwiak M Zhang Y Protogerou AD Millasseau S Non-invasive estimation of central systolic blood pressure by radial tonometry: a simplified approach. J Pers Med. (2023) 13(8):1–10. 10.3390/jpm13081244 PMC10455683 37623496

[56] Papaioannou TG Karageorgopoulou TD Sergentanis TN Protogerou AD Psaltopoulou T Sharman JE Accuracy of commercial devices and methods for noninvasive estimation of aortic systolic blood pressure a systematic review and meta-analysis of invasive validation studies. J Hypertens. (2016) 34(7):1237–48. 10.1097/HJH.0000000000000921 27136312

[57] Aguado-Sierra J Alastruey J Wang JJ Hadjiloizou N Davies J Parker KH. Separation of the reservoir and wave pressure and velocity from measurements at an arbitrary location in arteries. Proc Inst Mech Eng H. (2008) 222(4):403–16. 10.1243/09544119JEIM315 18595353

[58] Davies JE Baksi J Francis DP Hadjiloizou N Whinnett ZI Manisty CH The arterial reservoir pressure increases with aging and is the major determinant of the aortic augmentation index. Am J Physiol Heart Circ Physiol. (2010) 298(2):580–6. 10.1152/ajpheart.00875.2009 PMC2822572 20008272

[59] Baksi AJ Treibel TA Davies JE Hadjiloizou N Foale RA Parker KH A meta-analysis of the mechanism of blood pressure change with aging. J Am Coll Cardiol. (2009) 54(22):2087–92. 10.1016/j.jacc.2009.06.049 19926018

[60] Kass DA Shapiro EP Kawaguchi M Capriotti AR Scuteri A deGroof RC Improved arterial compliance by a novel advanced glycation end-product crosslink breaker. Circulation. (2001) 104(13):1464–70. 10.1161/hc3801.097806 11571237

[61] Tan E Algar S Corrêa D Small M Stemler T Walker D. Selecting embedding delays: an overview of embedding techniques and a new method using persistent homology. Chaos. (2023) 33(3):032101. 10.1063/5.0137223 37003815

